# Sodium bicarbonate to the ROSCue: a case report on how sodium bicarbonate successfully resuscitated a patient in cardiac arrest from suspected quetiapine and trazodone overdose

**DOI:** 10.1093/ehjcr/ytaf546

**Published:** 2025-10-27

**Authors:** Madhumita Kolluri, Jennifer Callaghan, Joel Wilken, Jason Gluck

**Affiliations:** Department of Internal Medicine, University of Connecticut Health, 263 Farmington Avenue, Farmington, CT 06030-1235, USA; Department of Cardiology, Hartford Healthcare, 85 Seymour Street Suite 603, Hartford, CT 06106, USA; Department of Internal Medicine, Hartford Healthcare, 80 Seymour Street, Hartford, CT 06102, USA; Department of Cardiology, Hartford Healthcare, 85 Seymour Street Suite 603, Hartford, CT 06106, USA

**Keywords:** Case report, Sodium bicarbonate, Cardiac arrest, Sodium channel blockade, Trazodone, Quetiapine

## Abstract

**Background:**

Per ACLS algorithms (Class III, LOE B), sodium bicarbonate (SB) use is indicated for cardiac arrest with underlying hyperkalaemia, tricyclic antidepressant (TCA) overdose, or pre-existing severe metabolic acidosis, with routine use of SB being discouraged due to concerns raised in animal studies. However, SB is a potent antidote for sodium channel blockade (SCB) in drug toxicities beyond TCA toxicity and should be considered more broadly.

**Case summary:**

We present a 50-year-old man who suffered an out-of-hospital cardiac arrest with a wide-complex bradycardia (QRS 184 ms) due to suspected quetiapine (atypical antipsychotic) and trazodone (serotonin modulator) overdose. After SB administration, the QRS narrowed to 142 ms and ROSC was immediately achieved.

**Discussion:**

Trazodone, a popular sleep medication, rarely leads to cardiac arrhythmias, and secondary cardiac arrest is exceptionally rare. Nonetheless, both trazodone and quetiapine can cause SCB in toxic doses. Intravenous SB can rapidly overcome medication-induced SCB and narrow a prolonged QRS and/or QTc interval. Although the role of SB in cardiac arrest remains limited, SB in SCB toxicity in TCA overdose *and* in other drug toxicities can be life-saving. SB should be considered in patients with wide QRS and suspected drug overdose.

Learning points

- To demonstrate that trazodone can cause cardiac arrhythmias in toxicity due to sodium channel blockade.
- To recognize sodium bicarbonate’s efficacy in treating arrhythmias caused by medication-induced sodium channel blockade.

## Introduction

Medication overdose is a leading cause for ED visits across the world, and intentional/accidental psychiatric medication overdoses are especially common.^1^ Antipsychotics and antidepressants can lead to fatal cardiac arrhythmias through sodium channel blockade (SCB), typically presenting with widening of the QRS complex, lengthening of the QT interval, new right axis deviation, bradyarrhythmia, ventricular tachycardia, ventricular fibrillation, and/or torsade des pointes.^2^

The 2010 AHA guidelines initially outlined the role of sodium bicarbonate (SB) in TCA overdose, and SCB in TCA toxicity has been widely studied. However, the 2020 AHA guidelines have since expanded their scope and now acknowledge the benefit of SB in other sodium channel blocker overdoses as well.^1–4^ In this case report, we discuss how i.v. SB successfully resuscitated a patient in cardiac arrest with an initial wide-complex bradycardia secondary to quetiapine and trazodone overdose.

## Summary figure

**Figure:**
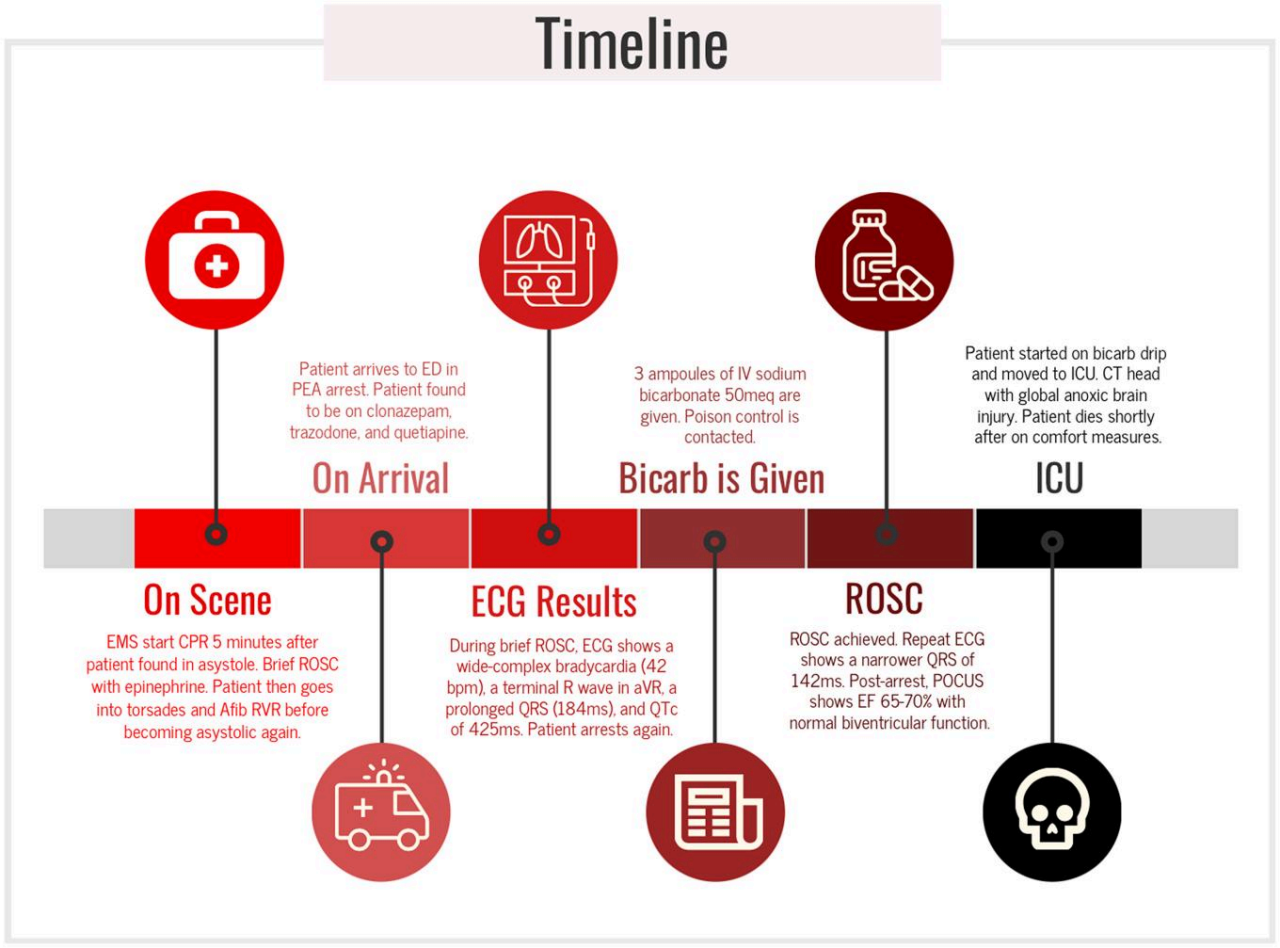


## Case presentation

**Figure 1:**
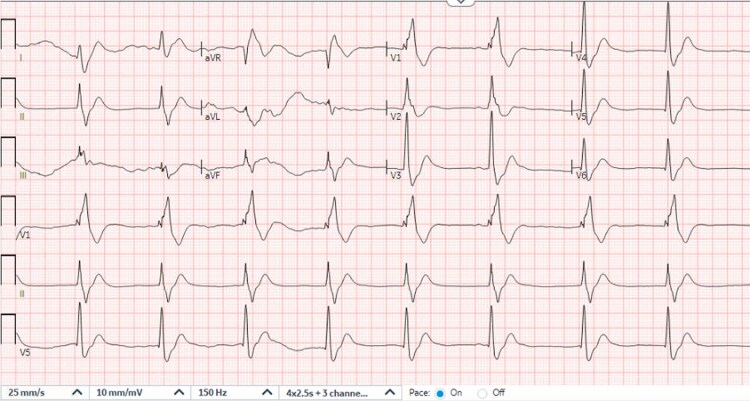
Initial ED ECG during brief ROSC: HR 53, QRS 184 ms, QTc 425 ms, wide-complex bradycardia.

A 50-year-old male was brought to the ED in cardiac arrest. The patient was in a taxi when he developed jerk-like movements and became unresponsive. Paramedics found the patient in asystole 5 min later and began CPR. Epinephrine was administered four times before ROSC, but the patient then went into polymorphic ventricular tachycardia and rapid atrial fibrillation before becoming asystolic again. In the ED, the patient was in PEA arrest and received magnesium sulphate, calcium gluconate, and more epinephrine. He briefly achieved ROSC and a 12-lead ECG (*Figure 1*) showed a wide-complex bradycardia (42 b.p.m.) with a terminal R wave in aVR, prolonged QRS (184 ms), and QTc of 425 ms before he went into PEA arrest again. His ABG showed pH <6.8, carbon dioxide >100 mmHg, SaO_2_ level of 76.1%, and bicarbonate too low to calculate. His creatinine was newly elevated at 1.7 mg/dl; and his lactic acid was 9.0 mmol/l. His creatinine kinase and high sensitivity troponin was 264 U/l and 34 ng/l, respectively. Urine toxicology was positive for cannabis.

**Figure 2:**
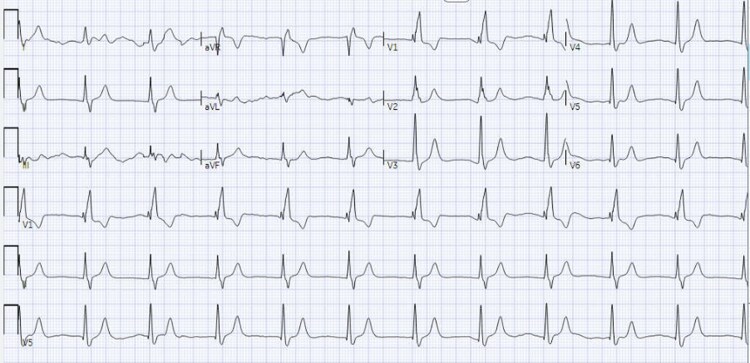
Subsequent ECG after 3 SB pushes: QRS 142, QTc 437, HR 67.

The patient did have a psychiatric history and had been taking quetiapine, trazodone, and clonazepam for schizophrenia and bipolar disorder. He also had history of prior suicidal ideation and medication experimentation. Hence, three ampules of 50meq i.v. SB were given for the suspected overdose and acidosis. Immediately, the patient achieved ROSC and narrowed his QRS to 142 ms (*Figure 2*). An SB drip was initiated, the patient was moved to the ICU, and Poison Control was contacted.

In the ICU, his ECG, electrolytes, and ABG (goal pH <7.55) were monitored every 2 h per Poison Control. He had mild hypokalaemia intermittently but no further arrhythmias. Despite being resuscitated for over an hour in the ED, his bedside echocardiogram showed normal biventricular function with a preserved ejection fraction. However, his high sensitivity troponin did increase to 2862 ng/l and gradually reached a peak of 9008 ng/l over the next 48 h. Given the prolonged arrest time, the lack of prior cardiac history, and no ischaemic ECG changes, it was determined that the patient had demand ischaemia and a very low likelihood of acute coronary syndrome.

**Figure 3:**
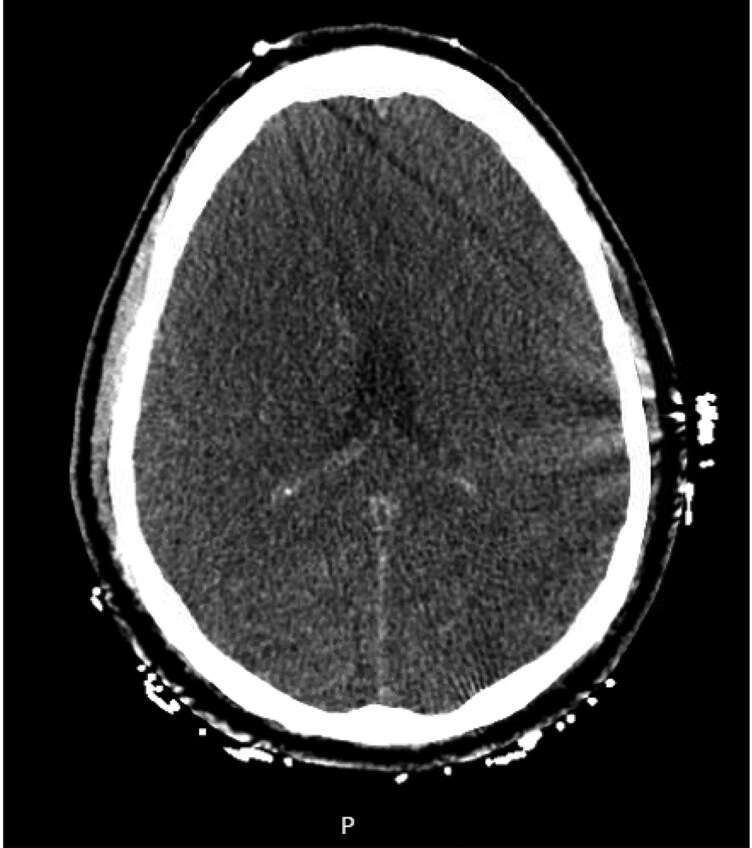
An image from the patient’s CT head showing loss of grey–white matter differentiation and sulcal effacement, consistent with global anoxic brain injury.

Neurologically, the patient’s initial exam post-ROSC showed unequal fixed and dilated pupils with absent brainstem reflexes. His non-contrast CT head scan the same day (*Figure 3*) showed extensive global cerebral anoxic injury with loss of grey to white matter differentiation throughout the entire cerebral hemispheres and focal cytotoxic oedema within the caudate nuclei and dorsomedial thalami. After close observation and family discussions regarding the patient’s poor neurological prognosis, he transitioned to comfort measures on day three of admission and died shortly after.

## Discussion

Despite controversy, SB remains one of the most widely used medications in cardiac arrest.^1,5^ Early ACLS guidelines (1970s–1980s) recommended SB during cardiac arrest, thinking SB improved the response to exogenous catecholamines by reducing metabolic acidosis severity from hypoxia.^1^ Animal studies then raised concern of SB leading to poor neurological outcomes in cardiac arrest due to increased systemic vascular resistance, hyperosmolality, hypernatremia, and increased intracellular acidosis.^1,5,6^

Out of 5589 out-of-hospital cardiac arrests, Chen *et al*.^7^ found that 15.1% of patients survived to hospital admission when SB was administered in the ED. Conversely, Wang *et al*.^5^ noted that only 11.7% of patients who received SB during in-hospital cardiac arrest survived to hospital discharge. Of the survivors, 5.6% demonstrated positive neurological recovery.^5^ After adjusting for confounders, a blood pH of 7.18 or less was found to be associated with a poor neurological outcomes.

Hence, causality between SB and lack of survival is yet to be established. Patients with longer duration of cardiac arrest are more likely to receive SB due to increasing metabolic acidosis, but two significant cofounders are longer cardiac arrest time and refractory acidosis, both of which are strongly associated with poor neurological outcomes.^5^ Perhaps the role (or lack thereof) of SB in neurological outcomes is biased by acidosis and/or cardiac arrest duration. This also prompts the question of when one should discontinue resuscitation efforts during cardiac arrest.

A wide range of medications can cause SCB during toxicity, not just TCAs. In a study by Simon *et al*.,^3^ diphenhydramine, amitriptyline, bupropion, quetiapine, nortriptyline, and cocaine were the most common agents to cause QRS prolongation out of 94 939 patients. Other case studies show that SB can rapidly overcome SCB in diphenhydramine and bupropion overdoses too.^8,9^

Exactly how SB reverses SCB is unknown, but it is thought to increase serum sodium concentration and/or change blood pH to improve acidosis.^1^ A prolonged QRS complex >0.10 s or a rightward shift on the ECG (a larger-than-expected S wave in lead I and R wave in lead aVr) are strongly suggestive of SCB.^10^ Untreated QRS prolongation is associated with seizures, ventricular dysrhythmias, and death.^3^ Notably, multiple logistic regression analysis have shown that an R wave of 3 mm or more in aVr is statistically significant for an increased risk of seizures or dysrhythmias.^10^

Trazodone, a serotonin modulator, rarely causes cardiac arrhythmias but is important to be aware of.^11^ High-dose trazodone inhibits all major cardiomyocyte ion channels (IKr, Iks, INa, and I Ca) through an especially high inhibitory potency human ether-a-go-go related gene (hERG). The hERG ion channel affects cardiac repolarization—blocking the hERG channel can prolong the QTc and lead to fatal ventricular dysrhythmias.^12^

In this case, multiple signs of SCB toxicity were present. First, the patient had recurrent cardiac arrhythmias in rapid succession despite a normal baseline ECG. This patient transitioned from asystole to torsades to rapid atrial fibrillation to PEA arrest. Torsades is common in drug toxicity.^1,3^ Presenting with asystole or PEA also suggests a non-cardiac aetiology of the arrest.^1,10,13^ Secondly, the patient had a wide-complex bradycardia with a right axis shift on the initial ECG. Both quetiapine and trazodone can cause SCB in toxicity;^14^ SCB often presents with a prolonged QRS morphology. The patient’s history of prior suicide attempts also raised suspicion for medication overdose. Finally, the rapid response to SB boluses with QRS reduction and ROSC further secured the diagnosis. Our case demonstrates how i.v. SB can effectively stabilize patients with acute trazodone and/or quetiapine overdose by overcoming excess SCB. However, the patient’s lack of bystander CPR and prolonged cardiac arrest of an hour led to a poor neurological outcome.

## Conclusion

Inadvertent sodium channel blocker overdose from medications other than TCAs are an underrecognized aetiology of cardiac arrest. Intravenous SB can rapidly overcome medication-induced SCB, narrow a prolonged QRS and/or QTc, and help maintain ROSC. Although SB use in cardiac arrest remains controversial, patients with cardiovascular instability and signs of SCB should receive SB. When administering SB, physicians should carefully monitor for hypokalaemia, hypocalcaemia, and alkalosis. Future prospective trials comparing QRS interval to mortality outcomes in patients with medication overdose could be key to better standardizing i.v. SB administration in emergent situations like cardiac arrest.

## Lead author biography


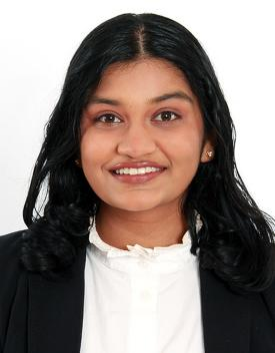


Dr Madhumita Kolluri is currently an internal medicine resident physician at the University of Connecticut. Originally from California, she pursued her medical education at the University of Plymouth, Peninsula Medical School and worked in the NHS as a foundation doctor before moving back to the United States to further pursue her career. Alongside her passions for research and student mentoring, Dr Kolluri hopes to become a cardiologist in the future.

## Data Availability

The data underlying this article are available in the article.
